# Home visits versus fixed-site care by community health workers and child survival: a cluster-randomized trial, Mali

**DOI:** 10.2471/BLT.23.290975

**Published:** 2024-06-25

**Authors:** Jenny Liu, Emily Treleaven, Caroline Whidden, Saibou Doumbia, Naimatou Kone, Amadou Beydi Cisse, Aly Diop, Mohamed Berthé, Mahamadou Guindo, Brahima Mamadou Koné, Michael P Fay, Ari D Johnson, Kassoum Kayentao

**Affiliations:** a Institute for Health and Aging, University of California, San Francisco, United States of America (USA).; b Institute for Social Research, 426 Thompson Street, University of Michigan, Ann Arbor, MI 48103, USA.; c Muso, SEMA, Bamako, Mali.; d Ministère de la Santé et du Développement Social, Bamako, Mali.; e National Institute of Allergy and Infectious Disease, Rockville, USA.; f Department of Medicine, University of California, San Francisco, USA.; g Malaria Research and Training Centre, University of Science, Technic and Technologies of Bamako, Bamako, Mali.

## Abstract

**Objective:**

To test the effect of proactive home visits by trained community health workers (CHWs) on child survival.

**Methods:**

We conducted a two arm, parallel, unmasked cluster-randomized trial in 137 village-clusters in rural Mali. From February 2017 to January 2020, 31 761 children enrolled at the trial start or at birth. Village-clusters received either primary care services by CHWs providing regular home visits (intervention) or by CHWs providing care at a fixed site (control). In both arms, user fees were removed and primary health centres received staffing and infrastructure improvements before trial start. Using lifetime birth histories from women aged 15–49 years surveyed annually, we estimated incidence rate ratios (IRR) for intention-to-treat and per-protocol effects on under-five mortality using Poisson regression models.

**Findings:**

Over three years, we observed 52 970 person-years (27 332 in intervention arm; 25 638 in control arm). During the trial, 909 children in the intervention arm and 827 children in the control arm died. The under-five mortality rate declined from 142.8 (95% CI: 133.3–152.9) to 56.7 (95% CI: 48.5–66.4) deaths per 1000 live births in the intervention arm; and from 154.3 (95% CI: 144.3–164.9) to 54.9 (95% CI: 45.2–64.5) deaths per 1000 live births in the control arm. Intention-to-treat (IRR: 1.02; 95% CI: 0.88–1.19) and per-protocol estimates (IRR: 1.01; 95% CI: 0.87–1.18) showed no difference between study arms.

**Conclusion:**

Though proactive home visits did not reduce under-five mortality, system-strengthening measures may have contributed to the decline in under-five mortality in both arms.

## Résumé

**Objectif:**

Évaluer l'impact qu'exercent des visites proactives à domicile par des agents de santé communautaires (ASC) formés à cet effet sur la survie infantile.

**Méthodes:**

Nous avons mené un essai randomisé non masqué à deux volets, en groupes parallèles, dans 137 localités des régions rurales du Mali. Entre février 2017 et janvier 2020, 31 761 enfants ont été recrutés au début de l'essai ou à leur naissance. Les localités ont bénéficié soit de soins de santé primaires prodigués par des ASC effectuant des visites régulières à domicile (intervention), soit d'une prise en charge sur un site fixe par des ASC (contrôle). Dans les deux volets, le ticket modérateur a été supprimé et les centres de soins de santé primaires ont fait l'objet d'améliorations au niveau du personnel et des infrastructures avant le lancement de l'essai. En nous fondant sur l'historique complet des naissances des femmes âgées de 15 à 49 ans, relevé chaque année, nous avons calculé les rapports de taux d'incidence (RTI) afin de mesurer les effets, selon l'intention de traiter et selon le respect du protocole, sur la mortalité chez les moins de cinq ans à l'aide de modèles de régression de Poisson.

**Résultats:**

Sur trois ans, nous avons observé 52 970 années-personnes (27 332 dans le volet d'intervention; 25 638 dans le volet de contrôle). Au cours de l'essai, 909 enfants du volet d'intervention et 827 enfants du volet de contrôle sont décédés. Le taux de mortalité des moins de cinq ans a baissé de 142,8 (IC de 95%: 133,3–152,9) à 56,7 (IC de 95%: 48,5–66,4) décès pour 1000 naissances vivantes dans le volet d'intervention tandis que, dans le volet de contrôle, il est passé de 154.3 (IC de 95%: 144,3–164,9) à 54,9 (IC de 95%: 45,2–64,5) décès pour 1000 naissances vivantes. Les estimations selon l'intention de traiter (RTI: 1,02; IC de 95%: 0,88–1,19) et selon le respect du protocole (RTI: 1,01; IC de 95%: 0,87–1,18) n'ont montré aucune différence entre les deux volets de l'étude.

**Conclusion:**

Bien que les visites proactives à domicile n'aient pas permis de réduire la mortalité des moins de cinq ans, les mesures de renforcement du système pourraient avoir contribué à la faire décroître dans les deux volets.

## Resumen

**Objetivo:**

Comprobar la influencia de las visitas domiciliarias proactivas de los agentes de salud comunitarios (ASC) con formación en supervivencia infantil.

**Métodos:**

Se realizó un ensayo con aleatorización de grupos, paralelo, desenmascarado y de dos grupos en 137 grupos de pueblos de las zonas rurales de Malí. De febrero de 2017 a enero de 2020, se inscribieron 31 761 niños al inicio del ensayo o al nacer. Los grupos de pueblos recibieron servicios de atención primaria prestados por ASC que realizaban visitas domiciliarias periódicas (intervención) o por ASC que prestaban atención en un lugar fijo (control). En ambos grupos, se eliminaron las tarifas a los usuarios y se mejoró la dotación de personal y la infraestructura de los centros de atención primaria antes del inicio del ensayo. A partir de los registros de nacimientos de mujeres de entre 15 y 49 años encuestadas cada año, se calcularon las tasas de incidencia (TI) de los efectos por intención de tratar y por protocolo sobre la mortalidad de menores de cinco años, mediante modelos de regresión de Poisson.

**Resultados:**

A lo largo de tres años, se observaron 52 970 personas-año (27 332 en el grupo de intervención y 25 638 en el grupo de control). Durante el ensayo murieron 909 niños en el grupo de intervención y 827 en el grupo de control. La tasa de mortalidad de menores de 5 años disminuyó de 142,8 (IC del 95%: 133,3-152,9) a 56,7 (IC del 95%: 48,5-66,4) muertes por 1000 nacidos vivos en el grupo de intervención y de 154,3 (IC del 95%: 144,3-164,9) a 54,9 (IC del 95%: 45,2-64,5) muertes por 1000 nacidos vivos en el grupo de control. Las estimaciones por intención de tratar (TI: 1,02; IC del 95%: 0,88-1,19) y por protocolo (TI: 1,01; IC del 95%: 0,87-1,18) no mostraron diferencias entre los grupos de estudio.

**Conclusión:**

Aunque las visitas domiciliarias proactivas no redujeron la mortalidad de menores de cinco años, las medidas de refuerzo del sistema pueden haber contribuido al descenso de la mortalidad de menores de cinco años en ambos grupos.

## ملخص

**الغرض:**

اختبار تأثير الزيارات المنزلية الاستباقية التي يقوم بها العاملون في مجال الصحة المجتمعية (CHW)، والمدربون على الحفاظ على حياة الأطفال.

**الطريقة:**

قمنا بإجراء تجربة عشوائية جماعية متوازية، غير محجوبة في مجموعتين، في 137 مجموعة من القرى في الريف المالي. خلال الفترة من فبراير/شباط 2017 إلى يناير/كانون ثاني 2020، تم تسجيل 31761 طفلًا عند بدء التجربة أو عند الولادة. حصلت مجموعات القرى إما على خدمات الرعاية الأولية من العاملين في مجال الصحة المجتمعية الذين يقدمون زيارات منزلية منتظمة (التدخل)، أو نظرائهم من العاملين الذين يقدمون الرعاية في موقع ثابت (التحكم). وفي كلا المجموعتين، تم إلغاء رسوم الاستخدام، واستقبلت مراكز الرعاية الصحية الأولية فرقًا للعمل، وحصلت على تحسينات في البنية التحتية قبل بدء التجربة. باستخدام تواريخ الولادات على مدى الحياة لسيدات تتراوح أعمارهن بين 15 و49 عامًا يتم فحصهن سنويًا، قمنا بتقدير نسب معدل الإصابة (IRR) الخاصة بتأثيرات نية العلاج، وتأثيرات كل بروتوكول بمفرده، على وفيات الأطفال تحت سن الخامسة، باستخدام نماذج التحوف لبواسون.

**النتائج:**

على مدى ثلاث سنوات، قمنا بفحص 52970 شخصًا كل سنة (27332 في مجموعة التدخل؛ و25638 في مجموعة التحكم). وخلال التجربة، تعرض 909 طفلاً في مجموعة التدخل، و827 طفلاً في مجموعة المراقبة، للوفاة. وانخفض معدل وفيات الأطفال تحت سن الخامسة من 142.8 (بفاصل ثقة مقداره %95: 133.3 إلى 152.9) إلى 56.7 (بفاصل ثقة مقداره %95: 48.5 إلى 66.4) حالة وفاة لكل 1000 من المواليد الأحياء في مجموعة التدخل، ومن 154.3 (بفاصل ثقة مقداره %95: 144.3 إلى 164.9) إلى 54.9 (بفاصل ثقة مقداره %95: 45.2 إلى 64.5) حالة وفاة لكل 1000 من المواليد الأحياء في مجموعة التحكم. إن نية العلاج (نسب معدل الإصابة: 1.02؛ ‏بفاصل ثقة مقداره %95: 0.88 إلى 1.19)، والتقديرات لكل بروتوكول بمفرده (نسب معدل الإصابة: 1.01؛ ‏بفاصل ثقة مقداره %95: 0.87 إلى 1.18) لم تظهر وجود فرق بين مجموعتي الدراسة.

**الاستنتاج:**

على الرغم من أن الزيارات المنزلية الاستباقية لم تحد من معدل وفيات الأطفال تحت سن الخامسة، إلا أن إجراءات دعم النظام قد تكون أسهمت في خفض معدل وفيات الأطفال تحت سن الخامسة في كلا المجموعتين.

## 摘要

**目的:**

旨在考查训练有素的的社区卫生工作者 (CHW) 主动上门拜访对儿童存活率的影响。

**方法:**

我们在马里农村地区的 137 个集聚型村落中进行了一项双臂、平行、未设盲的整群随机试验。从 2017 年 2 月到 2020 年 1 月,共有 31,761 名儿童在试验开始或其出生时入组。在集聚型村落,社区卫生工作者通过定期上门拜访(介入组)或定点护理(对照组)来提供初级保健服务。在试验开始之前,即已取消了两个组的使用费,并且初级保健中心也进行了人员配备和基础设施改善。利用对 15 至 49 岁女性的终生生育史年度调查,我们使用泊松回归模型估计了意向性治疗和符合研究方案对 5 岁以下儿童死亡率所产生影响的发生率比值 (IRR)。

**结果:**

在三年的时间里,我们观察了 52,970 人年(其中介入组为 27,332 人年;对照组为 25,638 人年)。试验期间,介入组有 909 名儿童死亡,而对照组的儿童死亡人数为 827 名。介入组五岁以下儿童的死亡率从每 1,000 例活产中死亡 142.8 例(95% CI:133.3-152.9)下降到 56.7 例(95% CI:48.5-66.4),对照组的该比率从每 1,000 例活产中死亡 154.3 例(95% CI:144.3-164.9)下降到 54.9 例(95% CI:45.2-64.5)。对意向性治疗(IRR:1.02;95% CI:0.88-1.19)和符合研究方案(IRR:1.01;95% CI:0.87-1.18)所产生影响的估计结果显示两个研究组之间没有差异。

**结论:**

虽然主动上门拜访没有降低五岁以下儿童的死亡率,但采取加强的系统性措施仍有可能降低两个组内五岁以下儿童的死亡率。

## Резюме

**Цель:**

Проверить влияние профилактических вызовов квалифицированных работников здравоохранения на дому на выживаемость детей.

**Методы:**

Было проведено открытое параллельное кластерное рандомизированное исследование с двумя группами в 137 деревенских кластерах в сельской местности Мали. С февраля 2017 года по январь 2020 года в начале исследования или при рождении был зарегистрирован 31 761 ребенок. Сельские кластеры получали услуги первичной медико-санитарной помощи либо от общинных медицинских работников, регулярно посещавших их на дому (вмешательство), либо от общинных медицинских работников, оказывавших помощь в фиксированном месте (контроль). В обеих группах была отменена плата за услуги, а центры первичной медико-санитарной помощи были укомплектованы персоналом и улучшили инфраструктуру до начала проведения испытания. Используя истории рождений за всю жизнь женщин в возрасте от 15 до 49 лет, ежегодно проходящих обследование, авторы выполнили оценку коэффициентов частоты заболеваемости (IRR) для влияния на смертность детей в возрасте до пяти лет по методу данных по всем пациентам, начавшим исследование, и по протоколу с использованием моделей регрессии Пуассона.

**Результаты:**

За три года наблюдениями было охвачено 52 970 человеко-лет (27 332 в группе вмешательства; 25 638 в группе контроля). За время исследования умерло 909 детей в группе вмешательства и 827 детей в группе контроля. Уровень смертности детей в возрасте до пяти лет снизился с 142,8 (95%-й ДИ: 133,3–152,9) до 56,7 (95%-й ДИ: 48,5–66,4) смерти на 1000 живорожденных в группе вмешательства и с 154,3 (95%-й ДИ: 144,3–164,9) до 54,9 (95%-й ДИ: 45,2–64,5) смерти на 1000 живорожденных в группе контроля. Оценка по методу подсчета данных по всем пациентам, начавшим исследование (IRR: 1,02; 95%-й ДИ: 0,88–1,19), и по протоколу (IRR: 1,01; 95%-й ДИ: 0,87–1,18) не выявила различий между группами исследования.

**Вывод:**

Хотя профилактические вызовы врачей на дом не привели к снижению смертности детей в возрасте до пяти лет, меры по укреплению системы, возможно, способствовали снижению смертности детей в возрасте до пяти лет в обеих группах.

## Introduction

Despite recent global declines, under-five mortality remains high in many of the poorest countries.^1^^,^^2^ Barriers to timely quality care, including user fees, distance to facilities and the availability of trained health workers and medical supplies, hinder progress in further reducing morbidity and mortality.^3^^,^^4^

Care provided by community health workers (CHWs) can improve access to health services and treatment adherence, and reduce disease-specific and all-cause mortality.^5^^–^^7^ However, CHW interventions can yield varying impacts,^7^^–^^12^ attributable to differences in intervention design and implementation.^13^ In particular, how CHW services should be optimized to overcome barriers to care is unclear, including distance to care.^14^^,^^15^ Proactive case detection via systematic home visits may improve timely access to care and reduce mortality by bringing services directly to patients’ homes, although the certainty of existing evidence is very low.^15^ Because patients may face delays in reaching care even within their communities,^16^ we hypothesize home visits will increase the speed at which patients receive care, resulting in reduced under-five mortality.

This study aims to analyse the effect of proactive case detection via home visits for reducing under-five mortality compared to fixed, site-based services delivered by professional CHWs integrated into the public sector health system in rural Mali.^17^ In 2018, the under-five mortality was 101 deaths per 1000 live births, and sub-national rates were as high as 152 deaths per 1000 live births in the country.^18^^,^^19^ We refer to all CHWs in both arms as professional because they were trained, paid, supervised and received regular supplies to conduct their work. We report the estimated intention-to-treat and per-protocol effects of the intervention on under-five mortality. We also compare under-five mortality in the pre-trial and trial periods across all clusters.

## Methods

### Study design

The Trial of Proactive Community Case Management to Reduce Child Mortality is a two arm, parallel, unmasked cluster randomized controlled trial testing the effectiveness of proactive case detection home visits (intervention) versus a passive workflow (control) delivered by CHWs. The trial was registered with ClinicalTrials.gov (NCT02694055) on 26 February 2016. Additional details are available in the published trial protocol.^17^ The trial was conducted over a three-year period from February 2017 through January 2020 in seven (Dimbal, Doundé, Ende, Kanibozon, Koulongon, Lessagou and Soubala) of 22 health catchment areas in the Bankass district in central Mali. Each catchment area is served by a public primary health centre. A public secondary referral hospital is located 35 km outside the study area. To our knowledge, no private sector providers operated in the study area during the trial period. At baseline, about 100 000 people were residing in the study area, and they were more impoverished than the average Malian household.^20^ The area had a high under-five mortality rate (152.6 deaths per 1000 live births) and lower rates of health-care utilization for acute illnesses among children younger than 5 years than the national average.^14^^,^^18^^,^^19^

The trial was powered to detect a 25% relative difference (*α* = 0.05, two-tailed test) in the incidence rate of under-five mortality between study arms; full details of the power calculations are available in the published trial protocol.^17^

Information about the trial pilot site and protocol deviations and amendments is available on Muso’s website.^21^

### Participants

All individuals in the study area, regardless of residency status, were able to receive health services from study CHWs or at referral primary health centres. All women of reproductive age (15–49 years) who were permanent residents (defined as residing in the area for at least six months with no other primary residence) and reported no plans to move away from the study area in the next three years were eligible to participate in annual household surveys assessing primary and secondary trial endpoints.

### Randomization and masking

After mapping all settlements within the study area, we defined 137 clusters as a grouping of villages and/or hamlets less than 1 km apart and at least 1 km from the next nearest grouping of villages and/or hamlets. We did not exclude any clusters from the trial. Trial statisticians stratified village-clusters along two dimensions: primary health centre catchment area and distance to the nearest primary health centre (< 1 km, 1–5 km and > 5 km). We chose 5 km as the distance aligns with national guidelines for deploying CHWs in communities situated more than 5 km from a health facility.^22^ An investigator who was not involved in study implementation, randomly assigned village-clusters within each strata to one of the arms, using a computer-generated random allocation. We masked trial statisticians to cluster allocation until the end of the trial and unmasked only after approval by the trial’s independent data safety and monitoring board. The original randomization scheme included 15 strata, with all villages less than 1 km from a primary health centre grouped into a single stratum. However, the randomization scheme implemented included 21 strata, with each village less than 1 km grouped in its own strata (online repository).^23^ The trial data analysis follows the randomization assignment as implemented by and verified with the field team, as recommended and approved by the data safety and monitoring board.

### Health-care provision

As per Mali’s national community health strategy, trained CHWs offered a comprehensive package of community-based primary care services from a fixed site in the community.^22^ The details of the procedure are described in Box 1. The study protocol includes additional information about the intervention and activities in the control arm.^17^

Box 1 **Procedures of the cluster-randomized trial Proactive Community Case Management to Reduce Child Mortality, Mali, 2017–2020**

Before the trial, CHWs in both arms received one month of health-related training. CHWs in the intervention arm received additional training related to the home visiting aspect of their work. To maintain equipoise, control arm CHWs provided health promotion, preventive and curative services to patients who sought care from the CHW at a fixed site in their assigned community. In most clusters, CHWs provided services from a fixed site within the village-cluster, separate from their homes. However, in clusters without fixed sites, CHWs offered services directly from their own homes. In the seven village-clusters that had a primary health centre in their village, CHWs still offered their services from a separate fixed site. The average distance from a household to the nearest CHW fixed site was less than 1 km in both arms.

CHWs in both arms worked the same total number of hours, approximately four hours per day, 6 days per week. To align with the national strategy, one CHW provided care for approximately 700 people. At least one CHW was stationed in a cluster, and supervisors conducted monthly visits to CHWs during the trial. CHWs were paid a monthly salary of 40 000 CFA (approximately US$ 71 in August 2018, the mid-point of the trial period), the same salary set by Malian health ministry for government-paid CHWs.

In January 2017, before the trial launch, participating primary health centres underwent systems strengthening measures. These measures involved removing all user fees and enhancing staffing and training, and improving equipment and infrastructure. Primary health centres are staffed by nurses, midwives and/or physicians.

***Services provision***

CHWs in the intervention arm offered the same set of services during proactive case detection visits to all households in their jurisdiction, with the goal of visiting each household at least twice per month. When CHWs in the intervention arm were not conducting proactive home visits, they provided services from fixed community sites in their cluster that were equivalent to control arm fixed sites.

There was no difference in the services or treatments available at the fixed CHW sites in the intervention versus control arms. CHWs provided community-based case management of malaria, diarrhoea, and pneumonia for children aged 2 to 59 months, and community-based case management of moderate acute malnutrition for children aged 6 to 59 months. For children younger than 5 years, CHWs received a standardized written tool to guide decisions about referrals, including a list of danger signs that required immediate referral to the primary health centre (difficulty breathing, seizure or convulsions). If the CHW found one or more danger signs, they accompanied the child immediately to the primary health centre using an available mode of transportation (e.g. a motorcycle) or called the primary health centre ambulance. If the CHW found no danger signs, they reviewed a list of signs or symptoms requiring referral. If one or more referral signs are found, the CHW accompanies the child immediately to the primary health centre if possible, or provides the parent with a referral sheet containing sociodemographic, clinical and laboratory parameters recorded for the child. Before accompaniment or referral to the primary health centre, the CHW measured the child’s temperature. If the temperature was elevated (≥ 37.5 °C), the CHW administered paracetamol. For symptomatic children aged 2 to 59 months, CHWs performed a malaria rapid diagnostic test (histidine-rich protein-2 antigen assay). If the test was positive, the CHW administered artemisinin-based combination therapy (arthemeter-lumefantrine). For children aged 2 to 59 months with diarrhoeal disease and no danger signs, CHWs administered oral rehydration therapy and zinc. For children aged 2 to 59 months with acute respiratory symptoms, CHWs assessed children for pneumonia by counting breaths. For children with associated symptoms and a respiratory rate of 50 breaths per minute for children up to 12 months and more than 40 breaths per minute for children 12–59 months, the CHW gave the child amoxicillin. CHWs examined babies younger than 2 months who exhibited any symptoms or were reported sick according to their caregiver for danger signs, and if present, they were immediately accompanied to the primary health centre. If no danger signs were present, they were referred to the primary health centre. CHWs were not trained in other aspects of newborn care, and CHWs in the control arm did not make home visits to provide home-based newborn care.

Newborns and infants younger than 6 months who were not gaining weight according to their caregiver were examined for danger signs; if none were present, they were referred to the primary health centre. CHWs measured the mid-upper arm circumference of children aged 6 to 59 months suspected to have acute malnutrition. CHWs offered children with mid-upper arm circumference of 11.5 to 12.5 cm (yellow zone) therapeutic food, albendazole and vitamin A. CHWs referred children in the red zone (< 11.5 cm) to the primary health centre.

CHWs referred children older than 5 years and adults who reported illness, patients requiring higher-level care and pregnant women for antenatal, delivery, and postnatal care to the participating primary health centres. CHWs did not offer services at a primary health centre before or during the trial.

CHW: community health worker; US$: United States dollar.

### Survey

We conducted household panel surveys at baseline (December 2016–January 2017), and after 12 months (February–March 2018), 24 months (March–May 2019) and 36 months (February–April 2020). All households in the study area were eligible to participate in this survey. Female interviewers, who were not a resident of the study area, administered the surveys to consenting (18 years or older) or assenting (15–17 years) women of reproductive age (at enrolment) at their homes. The survey instrument was adapted from the Mali Demographic and Health Survey questionnaire, encoded in Open Data Kit (Get ODK Inc. San Diego, United States of America) and loaded onto mobile tablets (Tecno Mobile, Shenzhen, China; or Samsung, Suwon, Republic of Korea) for use by interviewers. To obtain distance to the nearest primary health centre, we used a geographic information system to get the locations of each primary health centre and each concession (i.e. extended family grouping of households) at the time of enrolment.

Each survey included a household roster and modules on sociodemographic characteristics, reproductive and maternal health, and recent illness and health-care utilization among children younger than 5 years. The sex of each child was reported by the mother. At follow-up surveys, respondents reported their lifetime birth histories and the number of CHW home visits their household received in the preceding month. We updated household rosters at each survey round to identify new members (due to births, migration, marriage or adoption) and those absent due to migration or death. At each time point, we invited newly eligible women (reaching reproductive age or arriving in study area) to participate. In all surveys, we made up to three attempts to contact each eligible household and woman.

### Outcomes

The trial’s primary endpoint is all-cause under-five mortality. We obtained information about children’s vital status from birth histories in each follow-up survey. Children are at risk of death beginning at their date of birth, the start of the trial for those born before the baseline survey, or the interview date in which they are first reported as present in the household. Children are lost to follow-up when the household could not be located in a subsequent household survey or no household member was available to participate. Children are right censored at the end of the trial, their fifth birthday or when lost to follow-up, depending on which occurred first.

### Statistical analysis

Details of our analytical approach and sample size calculations are given in the trial statistical analysis plan (online repository).^23^ All results are presented using an intention-to-treat approach unless otherwise noted. We systematically tested observable cluster and individual characteristics at trial start for differences by arm, accounting for the clustered nature of the data. We calculated crude death rates as the number of deaths among children younger than 5 years per 1000 person-years of exposure. We estimated the under-five mortality rate as the probability of dying between birth and the fifth birthday per 1000 live births. To estimate the under-five mortality rate for the three-year period before (February 2014 through January 2017) and during the study period, we used a life table approach with lifetime birth history data to estimate mortality probabilities in eight age segments (0, 1–2, 3–5, 6–11, 12–23, 24–35, 36–47 and 48–59 months) to account for non-proportional differences in age-specific mortality rates across early childhood.^24^

We used a time updated Poisson regression model at the child-month level to estimate the effect of the intervention on the incidence rate ratio (IRR) of under-five mortality using an intention-to-treat approach (primary effect analysis). We adjusted for non-constant risk of death in early childhood by controlling for age (months) and differential of risk of under-five mortality by sex by controlling for sex of the child. Models also adjusted for household distance to the nearest primary health centre (≤ 5 km versus > 5 km). All models used robust standard errors adjusted for clustering at the village-cluster level to account for correlation among observations at the unit of randomization. We report the intention-to-treat effects as the IRR between intervention and control arms with 95% confidence intervals (CI).

We also estimated the per-protocol effect of the intervention. For the intervention arm, we defined treatment adherence as receiving two or more home visits from a CHW in the month preceding the survey for all years in which the household was enrolled.^17^ In the control arm, we defined adherence as receiving no home visits in the preceding month in any year in which the household was enrolled. We estimated stabilized inverse probability weights for protocol deviation using pooled logistic regression fit by maximum likelihood, where the denominator included individual, household and village-level covariates.^25^^,^^26^ We then estimated the IRR of under-five mortality using the time updated Poisson regression models described above with stabilized inverse probability weighting.

We examined the possibility of heterogeneous treatment effects based on intention-to-treat and per-protocol analyses. We interacted the intervention arm indicator with subgroup indicators defined at baseline, including distance to primary health centre, village-cluster population size and household wealth. We conducted all analyses in Stata Version 17.1 (StataCorp LCC, College Station, USA).

The methods for the various sensitivity analyses we conducted are available in the online repository.^23^

### Ethical approvals and trial oversight

The Ethics Committee of the Faculty of Medicine, Pharmacy and Dentistry at the University of Bamako, Mali, approved the trial (2016/03/CE/FMPOS). Secondary analysis of trial data was exempted from ethical review by the University of California, San Francisco, United States (Ref: 154824) and approved by the observational and interventions research ethics committees at the London School of Hygiene & Tropical Medicine, London, England (Ref: 13832). All participants gave written informed consent for each annual household survey.

The trial was externally monitored by Pharmalys, Borehamwood, England. An independent data safety and monitoring board oversaw participant safety and evaluation of interim results. Since 2018, the study area experienced a marked increase in armed conflict-related events and fatalities. Subsequent protocol amendments and deviations to assure the safety of participants, providers and study personnel were reviewed by Pharmalys and the independent board and approved by the governing ethics committee (online repository).^23^

## Results

**Fig. 1:**
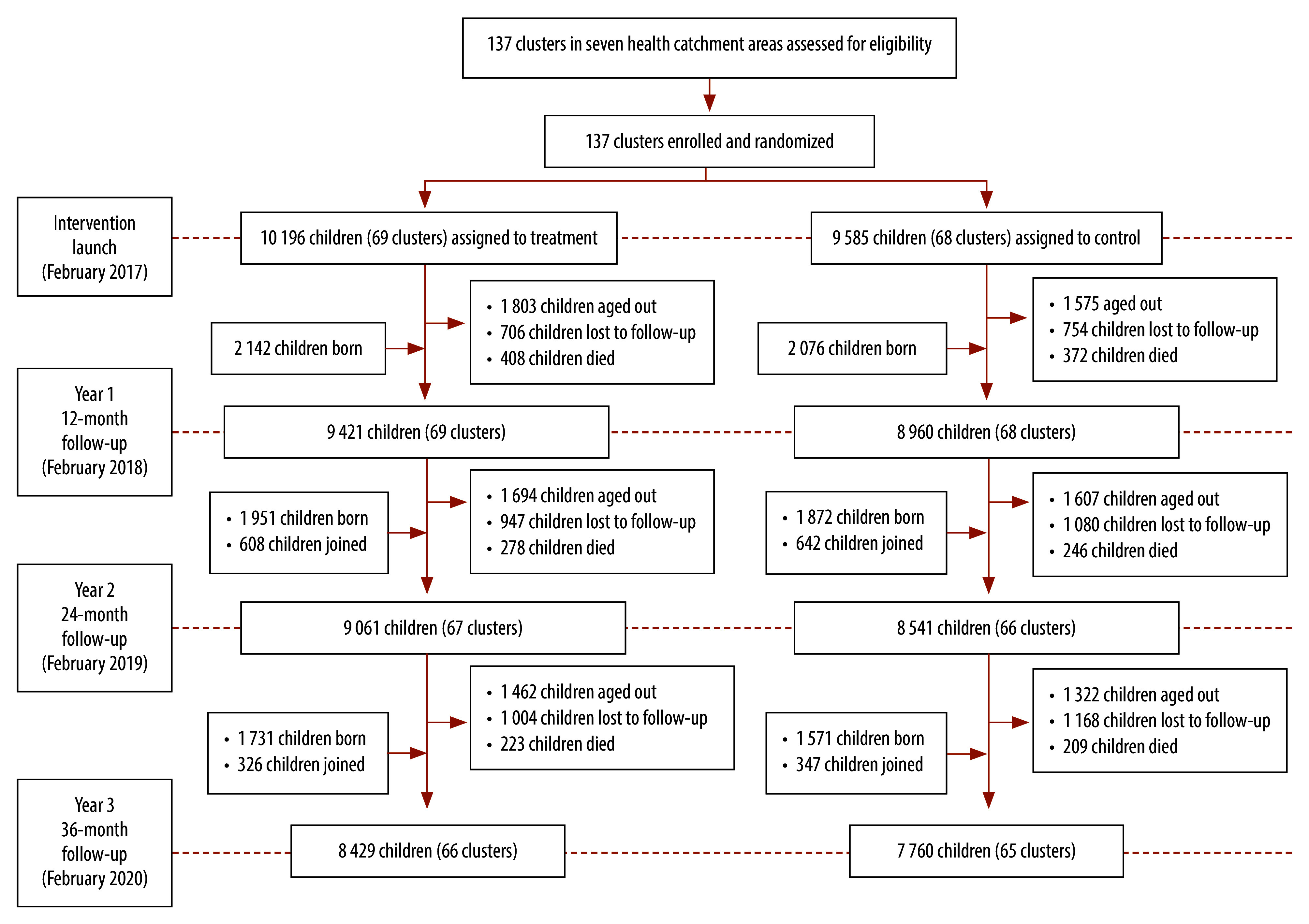
Flowchart of participants of the cluster-randomized trial on proactive home visits by community health workers, Mali, 2017–2020

We enrolled and randomized 137 village-clusters. Due to armed conflict, six clusters were lost to follow-up during the trial (online repository).^23^ Fig. 1 shows the flowchart of the trial and Table 1 presents number of children, by sex, included at different stages of the trial. In summary, over the trial period, 31 587 children were enrolled (16 248 in intervention arm and 15 339 in control arm, totalling 52 970 person-years (635 644 person-months) of observation (27 332 in intervention arm and 25 638 in control arm).

**Table 1:** Number of children at different stages of the cluster-randomized trial Proactive Community Case Management to Reduce Child Mortality, Mali, 2017–2020

Variable	No.^a^										
	Total				Intervention arm				Control arm		
	Children	Male	Female		Children	Male	Female		Children	Male	Female
Enumerated at baseline	19 781	10 040	9 741		10 196	5 159	5 037		9 585	4 881	4 704
Enrolled	31 587	15 957	15 630		16 248	8 110	8 138		15 339	7 847	7 492
Person-years observed (months)	52 970 (635 644)	26 715 (320 586)	26 255 (315 058)		27 332 (327 991)	13 668 (164 025)	13 664 (163 966)		25 638 (327 991)	13 047 (156 561)	12 591 (151 092)
Mothers reporting on outcome	15 172	NA	NA		7 734	NA	NA		7 438	NA	NA
Deaths	1 736	916	820		909	485	424		827	431	396
Aged out of trial	9 463	4 805	4 658		4 959	2 511	2 448		4 504	2 294	2 210
Lost to follow up	5 659	2 837	2 822		2 657	1 305	1 352		3 002	1 532	1 470

NA: not applicable

^a^ Values represent number of children unless otherwise stated.

At the start of the trial, we did not identify any significant differences in characteristics of individuals across study arms (Table 2). For the three-year trial period, the crude death rates were similar across arms, 33.26 per 1000 person-years in intervention arm versus 32.36 per 1000 person-years in control arm. Deaths declined over the course of the trial in both arms: from 42.90 (year 1) to 25.71 per 1000 person-years (year 3) in the intervention arm; and from 41.52 (year 1) to 25.97 per 1000 person-years (year 3) in the control arm (Table 3).

**Table 2:** Individual-level characteristics at start of the cluster-randomized trial Proactive Community Case Management to Reduce Child Mortality, Mali, 2017–2020

Characteristic	No. (%)^a^		
	Intervention arm (10 196 children)	Control arm (9 585 children)	Total (19 781 children)
**No. of households^b^**	5 267 (50.83)	5 097 (49.17)	10 366 (100.0)
**Median no. of children younger than 5 years per household (SD)**	2.57 (1.38)	2.48 (1.30)	2.53 (1.34)
**Child’s age, in months**			
0–5	1 297 (12.72)	1 179 (12.30)	2 476 (12.52)
6–11	1 002 (9.83)	1 013 (10.57)	2 015 (10.19)
12–23	2 000 (19.62)	1 884 (19.66)	3 884 (19.64)
24–35	2 006 (19.67)	1 893 (19.75)	3 899 (19.71)
36–59	3 891 (38.16)	3 616 (37.73)	7 507 (37.95)
**Child’s sex**			
Male	5 159 (50.60)	4 881 (50.92)	10 040 (50.76)
Female	5 037 (49.40)	4 704 (49.08)	9 741 (49.24)
**Median household size (SD)**	9.97 (4.73)	9.78 (4.76)	9.88 (4.75)
**Mother has attended any school**	691 (6.78)	770 (8.03)	1 461 (7.39)
**Mother’s marital status**			
Single^c^	104 (1.02)	98 (1.02)	202 (1.02)
Married, monogamous	5 398 (52.94)	5 173 (53.97)	10 571 (53.44)
Married, polygynous	4 574 (44.86)	4 208 (43.90)	8 782 (44.40)
Missing	120 (1.18)	106 (1.11)	226 (1.14)
**Household wealth^d^**			
Poorest	1 758 (17.24)	1 515 (15.81)	3 273 (16.55)
Poor	1 835 (18.00)	1 905 (19.87)	3 740 (18.91)
Middle	2 039 (20.00)	1 966 (20.51)	4 005 (20.25)
Rich	2 175 (21.33)	2 125 (22.17)	4 300 (21.74)
Richest	2 389 (23.43)	2 074 (21.64)	4 463 (22.56)
**Cluster distance to health facility, in km**			
≤ 5	4 175 (40.95)	4 582 (47.80)	8 757 (44.27)
> 5	6 021 (59.05)	5 003 (52.20)	11 024 (55.73)
**Cluster population at baseline**			
< 700	2 873 (28.18)	3 287 (34.29)	6 160 (31.14)
≥ 700	7 323 (71.82)	6 298 (65.71)	13 621 (68.86)

^a^ Values represent no. (%) unless otherwise stated.

^b^ In this row, the denominator is 10 366 households.

^c^ Single refers to mothers never married, widowed or divorced.

^d^ We estimated household wealth using a principal component analysis of household ownership of durable goods, livestock and physical housing characteristics.

**Table 3:** Deaths among children younger than 5 years participating in a cluster randomized trial Proactive Community Case Management to Reduce Child Mortality, Mali, 2017–2020

Trial period	Intervention			Control		
	Deaths	Person-years	Deaths/1000 person-years	Deaths	Person-years	Deaths/1000 person-years
Year 1	408	9 511.17	42.90	372	8 959.08	41.52
Year 2	278	9 148.17	30.29	246	8 630.33	28.50
Year 3	223	8 673.25	25.71	209	8 048.33	25.97
Total	909	27 332.58	33.26	827	25 637.25	32.36

**Fig. 2:**
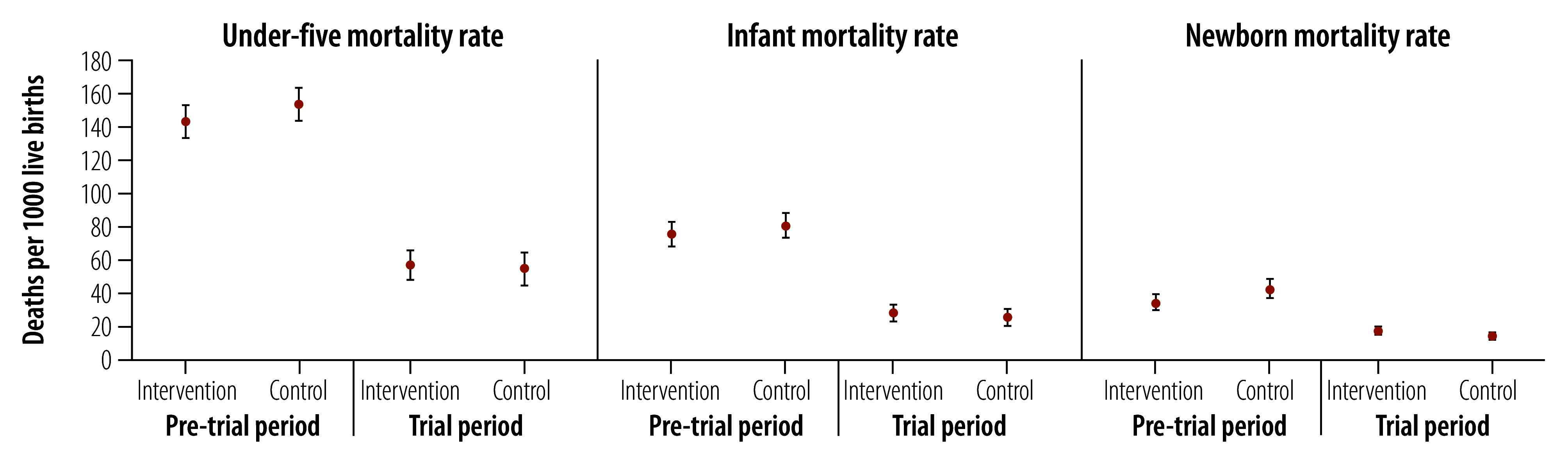
Under-five, infant and newborn mortality rates by arm in pre-trial versus trial periods, Mali, 2014–2020

Notes: under-five mortality refers to dying between birth and the fifth birthday; infant mortality refers to dying between birth and the first birthday; and neonatal mortality refers to dying between birth and the 28th day of life. The pre-trial period was between February 2014 and January 2017, and the trial period was between February 2017 and January 2020.

Across arms, the under-five mortality rate declined from 148.4 (95% CI: 141.5–155.7) deaths per 1000 live births over the three years before trial to 55.1 (95% CI: 48.6–62.4) deaths per 1000 live births over the trial period. We observed similar declines in the infant and newborn mortality rates in both arms. No rates differed by arm in the pre-trial or trial period. Under-five mortality rate declined from 142.8 (95% CI: 133.3–152.9) to 56.7 (95% CI: 48.5–66.4) deaths per 1000 live births in the intervention arm; and from 154.3 (95% CI: 144.3–164.9) to 54.9 (95% CI: 45.2–64.5) deaths per 1000 live births in the control arm (Fig. 2).

In the intention-to-treat analysis, the incidence rates of under-five mortality in the intervention and control arms were similar (Table 4; IRR: 1.02; 95% CI: 0.88–1.19). The incidence rates did not vary by sex, distance to primary health centre, cluster population size or household wealth (online repository).^23^ As expected, the incidence rate declined with increasing age of the children (online repository).^23^

**Table 4:** Estimated intention-to-treat and per-protocol effects of the intervention on under-five mortality^a^

Variable	IRR (95% CI)^b^	
	Intention to treat^c^	Per protocol^c,d^
**Trial arm**		
Intervention	1.02 (0.88–1.19)	1.01 (0.87–1.18)
Control	Reference	Reference
**Child’s age, in months**		
0	1161.20 (928.29–1452.54)	1197.49 (946.32–1515.32)
1–2	45.48 (32.88–62.91)	45.41 (32.78–62.91)
3–5	14.75 (10.83–20.08)	14.46 (10.48–19.97)
6–11	7.87 (5.84–10.59)	7.99 (5.89–10.91)
12–23	5.32 (4.17–6.78)	5.44 (4.21–7.04)
24–35	3.39 (2.64–4.36)	3.55 (2.74–4.61)
36–47	2.03 (1.55–2.66)	2.12 (1.60–2.82)
48–59	Reference	Reference
**Child’s sex**		
Female	0.91 (0.81–1.02)	0.91 (0.80–1.03)
Male	Reference	Reference

IRR: incidence rate ratio.

^a^ Analysis based on 52 970 person-years.

^b^ Comparing the incidence rate of under-five mortality in the intervention arm to the control arm.

^c^ Estimated via a time-updated Poisson regression adjusted for facility distance; robust standard errors adjusted for clustering at the village-cluster level.

^d^ Estimated with stabilized inverse probability of treatment weights.

Only 25.0% (4070/16 248) of children in the intervention arm met the per-protocol criteria of two or more home visits in the preceding month, while 73.5% (11 271/15 339) of children in the control arm met the criteria of no home visits in the preceding month (online repository).^23^ All interventions, and all but one control clusters, included children who met the per-protocol definition. Per-protocol estimates show no difference in mortality associated with intervention exposure (Table 4; IRR: 1.01; 95% CI: 0.87–1.18).

These results are robust to various sensitivity analyses, including those accounting for potential biases from missing data for children’s age, birth date and death date (online repository).^23^ Despite notable lost to follow-up, we find no differential entry or lost to follow-up by arm. We find no substantive difference in effect estimates when restricting the sample to children born at least 9 months after trial launch, that is, children who were exposed to the intervention in utero and whose trial entry was not conditional on survival to trial launch. Finally, conducting the intention-to-treat analysis at the village-cluster level yielded the same null effect as did individual-level specifications using Cox proportional hazard models.

## Discussion

Our three-year cluster randomized controlled trial to test the effectiveness of CHW home visits compared to community-based fixed-site care by CHWs in a context where user fees were removed, CHWs were stationed in all communities and health systems strengthening measures were deployed in both arms. The study did not show an attributable difference in all-cause under-five mortality between arms. However, compared to the period before the trial, we observed a substantial decline in the under-five mortality rate in both arms to a rate lower than for almost all other regions in Mali.^27^ The observed decline is notable given the onset of armed conflict in the study area, as such conflicts are associated with increases in under-five mortality.^28^ The onset of armed conflict and migration of participants resulted in losses to follow-up and moderate adherence.

The trial addresses a critical gap in the literature by providing rigorous evidence about the impact of CHW service organization on all-cause under-five mortality.^15^ Prior studies identifying positive effects of CHW home visits focused on disease- or period-specific effects. For example, home visits in the postpartum period reduced newborn mortality,^29^ and home visits for proactive malaria case detection and management led to increased treatment.^30^ When deciding about community health workflows, policy-makers must consider costs and benefits of CHW home visits for multiple outcomes. Analyses of trial secondary endpoints showed no difference between arms in the prevalence of diarrhoea, febrile illness or acute respiratory infections. However, at 12 months, children younger than 5 years in the intervention arm were more likely to promptly access health services than children in the control arm.^31^ By 24 and 36 months, there was no difference in health-care utilization by arm. Health-care utilization in both arms increased from a median of 19% at baseline to 52% at trial completion across all clusters despite the onset of armed conflict.^31^ The intervention also increased early initiation and uptake of antenatal care relative to the control arm, though the intervention did not affect facility delivery.^32^ Both antenatal care and facility delivery increased across arms relative to the pre-trial period. The presence of some intervention effects across trial arms suggests that the null effect on under-five mortality results from a lack of impact of CHW home visits compared to fixed-site care, rather than poor adherence. In future analyses, we will test spatial and dose–response relationships on under-five mortality.

The overall under-five mortality rate decline suggests that system-strengthening measures deployed in both arms, regardless of distance to the nearest primary health centre, could be more important for child survival than CHW service location. User fee removal and locating professional CHWs in communities were associated with increased health-care utilization and reduced under-five mortality in other studies, including in Mali.^9^^,^^33^^–^^39^ Before the trial, CHW services were inconsistently provided in communities 5 km or more from the nearest primary health centre. However, an analysis of pre-trial data showed significantly lower child health-care utilization among children in villages just 2 km from a primary health centre, relative to those living within 2 km.^14^ Addressing cost, distance and clinical capacity – key determinants of health-care utilization and under-five mortality^3^^,^^16^^,^^40^^,^^41^ – may have been particularly important in the context of armed conflict, which disrupts health-care delivery and access.^42^^–^^44^ To contextualize the trial results, this decline was greater than that observed nationally in Mali over the trial period, from 108.9 deaths per 1000 live births in 2017 to 99.7 deaths per 1000 live births in 2020.^27^ The presence of armed conflict may limit the generalizability of our findings to non-conflict settings. However, since such interventions are rare in conflict zones, our results can inform health-care design and delivery in similar contexts. Moreover, lessons from the trial may apply to rural areas with high under-five mortality rates, where many cannot afford health-care fees and face long distances to access care.^16^^,^^27^^,^^45^^,^^46^ There was no difference in the intervention’s effect on under-five mortality among boys versus girls.

Strengths of the trial include its sample size, rigorous measurement of endpoints and longitudinal design. Limitations include the potential for errors in annually following up participants in a highly mobile population; and lack of data on cause of death, in addition to the low adherence observed in the intervention arm and loss to follow-up in both arms. Estimates of pretrial under-five mortality rates may be subject to recall bias due to the three-year recall period. However, we do not expect that recall bias varies by arm. We cannot confirm the location of CHW visits as these measures are based on self-report. Finally, to maintain equipoise, we do not compare the intervention arm to a sample with no CHW. At the time of the trial in Mali, the standard of care dictated that CHWs be stationed at fixed sites in all villages located more than 5 km away from a primary health centre. This practice was reflected in both arms. Analysis of CHW mobile application data and programme costs will provide further insight into fidelity to protocols, quantify CHW services delivered, and characterize the dose–response relationship to health outcomes. Our process evaluation will further contextualize study results, including mechanisms of effect for systems strengthening measures.^47^

We found that proactive home visits by CHWs did not reduce under-five mortality compared to the same CHW services offered at a fixed community site, addressing a key question for policy-makers in low- and middle-income countries. The deployment of professional CHWs in all communities, the removal of user fees, and other system-strengthening measures benefiting patients in both arms may have contributed to the declines in under-five mortality. Further analyses of trial data will help identify the specific aspects that contributed to increased child survival.
